# Prevalence of trachoma within refugee camps serving South Sudanese refugees in White Nile State, Sudan: Results from population-based surveys

**DOI:** 10.1371/journal.pntd.0007491

**Published:** 2019-06-13

**Authors:** Angelia M. Sanders, Zeinab Abdalla, Belgesa E. Elshafie, Andrew W. Nute, Elizabeth F. Long, Nabil Aziz, Paul Weiss, E. Kelly Callahan, Scott D. Nash

**Affiliations:** 1 The Carter Center, Atlanta, Georgia, United States of America; 2 The Carter Center, Khartoum, Sudan; 3 National Program for Prevention of Blindness, Federal Ministry of Health, Khartoum, Sudan; 4 Department of Biostatistics and Bioinformatics, Rollins School of Public Health, Emory University, Atlanta, Georgia, United States of America; RTI International, UNITED STATES

## Abstract

**Background:**

The world is witnessing mass displacement of populations which could impact global efforts to eliminate neglected tropical diseases such as trachoma. On the African continent, South Sudan has experienced high levels of population displacement. Population based baseline trachoma surveys were conducted among refugee camps in two Sudanese localities hosting South Sudanese refugee populations to determine whether the SAFE strategy was warranted.

**Methodology/Principal findings:**

Cross-sectional, multi-stage, cluster-random surveys were conducted within refugee camps in each of two Sudanese localities, Al Salam and Al Jabalain. For survey sampling, multiple camps within each locality were combined to form the sampling frame for that locality. Household water, sanitation and hygiene indicators were assessed, and trachoma signs were graded by trained and certified graders. The prevalence of trachomatous inflammation-follicular (TF) in children aged one to nine years was 15.7% (95%CI: 12.1–20.2) in Al Salam and 10.6% (95%CI: 7.9–14.0) in Al Jabalain. The prevalence of trachomatous trichiasis (TT) in those 15 years above was 2.9% (95%CI: 1.8–4.8) in Al Salam and 5.0% (95%CI: 3.8–6.6) in Al Jabalain. The presence of water and sanitation was high in both survey units.

**Conclusion/ Significance:**

Sudan has made progress in reducing the prevalence of trachoma within the country; however, the presence of over one million refugees from a neighboring trachoma hyper-endemic country could impact this progress. These surveys were the first step in addressing this important issue. The results demonstrate that at least three years of mass drug administration with azithromycin and tetracycline is needed in addition to the provision of TT surgical services. Additionally, it highlights that non-endemic or formerly endemic localities may have to adopt new strategies to provide services for refugee populations originating from hyper-endemic regions to ensure elimination of trachoma as a public health problem for all populations.

## Author summary

Ministries of health in multiple countries have made progress in reducing the prevalence of trachoma, the leading cause of infectious blindness. With the increase in displaced populations throughout the world, the work of these national programs could be put at risk as formerly endemic or non-endemic districts now host large numbers of refugees from trachoma endemic regions. To properly respond, national programs must first assess the extent of the disease. We conducted baseline surveys in South Sudanese refugee camps located in two Sudanese localities to determine the prevalence of trachoma. These surveys showed that conducting prevalence surveys in refugee camp settings was possible. Trachoma was found to be present within the refugee camp population and programmatic interventions are required. The global community cannot expect to eliminate trachoma in the near future if displaced populations within countries and across country borders are not addressed.

## Introduction

Trachoma, the leading infectious cause of blindness, is caused by infection with the bacterium *Chlamydia trachomatis* [1]. Infection occurs through personal contact via hands, clothes, and flies that have been in contact with the infected discharge from the eyes or nose of an infected person. Repeated episodes of infection over many years can cause scarring of the inner eye lid and eventually lead to the eyelids turning inward and eyelashes rubbing the surface of the eye until blindness occurs. Blindness from trachoma is irreversible. The “SAFE” strategy: Surgery to correct misdirected eyelashes, Antibiotics to treat the infection within the body, Facial cleanliness to remove the discharge from the eyes and nose, and Environmental improvement through the building and use of latrines, is used by country programs to assist in eliminating trachoma as a public health problem [2].

Despite the progress that has been made by endemic countries to eliminate trachoma as a public health problem [3], the increasing displacement of large populations from trachoma endemic areas could impact global progress. The United Nations High Commissioner for Refugees (UNHCR) has stated that the world is currently witnessing the highest levels of displacement on record, with 68.5 million people categorized as forcibly displaced in 2017 [4]. This figure includes 40 million internally displaced people (IDPs) and 25.4 million refugees. Over 50% of current refugees are from three countries: Afghanistan, Syria, and South Sudan. Of the world’s refugees, 85% are hosted in countries designated as least developed countries. This displacement can impact national neglected tropical disease (NTD) programs, particularly trachoma, as ministries of health work to care for not only their own citizens but refugees as well. Ministries, supporting agencies and donors must determine how to include refugees when implementing trachoma interventions.

In January 2005, fighting between the Government of Sudan and the southern-based Sudan People’s Liberation Army ended after a Comprehensive Peace Agreement was signed. Following an election, South Sudan became an independent country in July 2011. On December 15, 2013 fighting between factions within the Government of South Sudan resulted in a protracted conflict, with the greatest military confrontations in Jonglei, Upper Nile and Unity states [5]. The conflict eventually led to increased food insecurity and caused much of the population to need food aid. Since December 2013, this complex emergency has resulted in 1.91 million IDPs and over 2.46 million refugees, making the South Sudanese refugee crisis the largest and fastest growing on the African continent [6,7]. Though efforts have been made at peace, the UNHCR continues to document new refugees arriving in neighboring countries such as Sudan [8].

**Fig 1:**
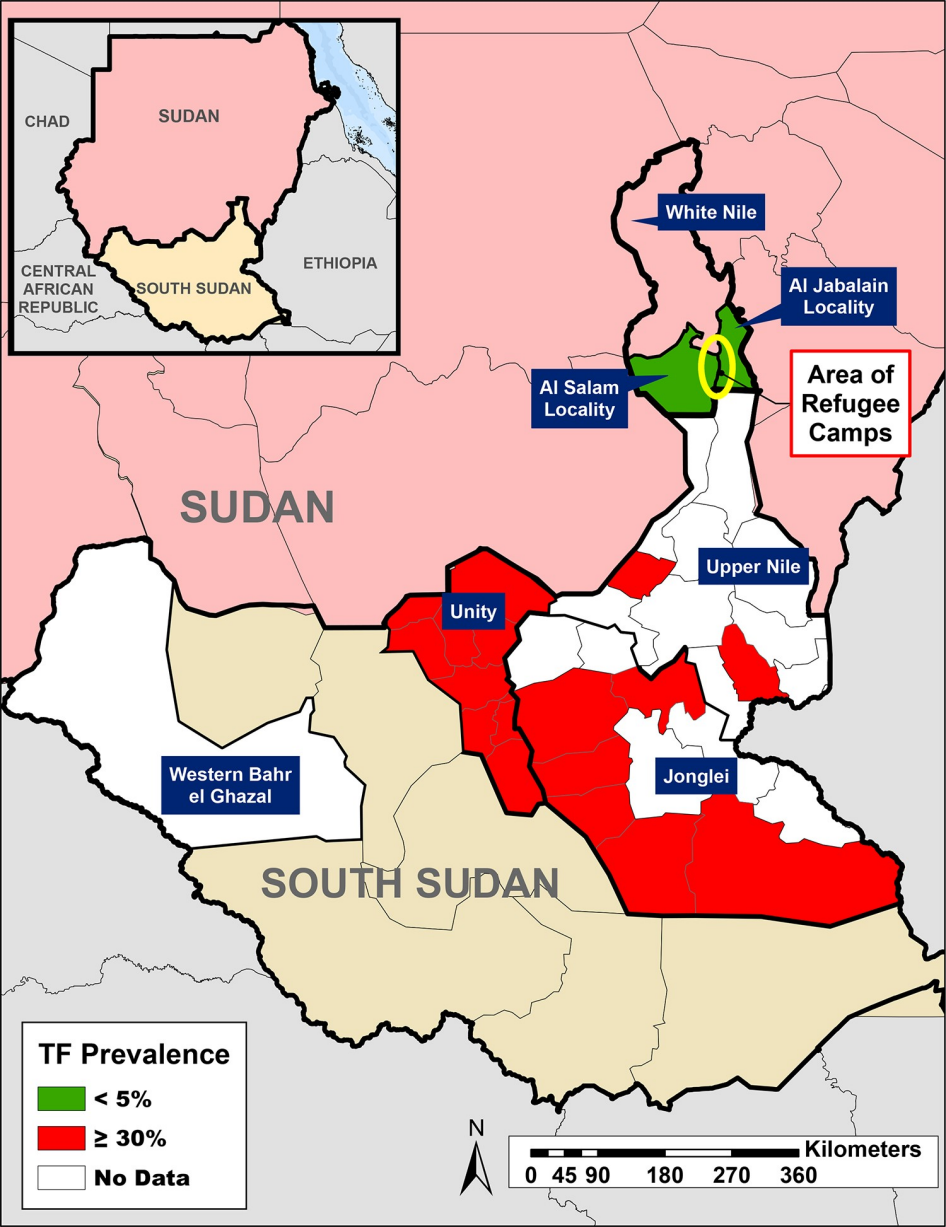
Location of Al Salam and Al Jabalain refugee camps in White Nile State, Sudan; location of refugee originating states in South Sudan and their documented TF prevalence in children 1–9 years. (Software: ArcGIS 10.6, ESRI; Shapefiles: gadm.org, retrieved/customized in 2014).

Both Sudan and South Sudan are endemic for trachoma [9–16]. Over one million South Sudanese fled to neighboring Sudan as refugees, with the majority settling in White Nile and Khartoum states [8]. Baseline surveys in White Nile state in 2009 showed that all localities (equivalent of a district) had below 5% trachomatous inflammation-follicular (TF) in children aged one to nine years, except for Al Jabalain locality which had a 6.4% TF prevalence [15]. Trachomatous trichiasis (TT) was at or below 0.2% in those 15 years and above in all but three localities (Al Getina, Al Salam, and Al Jabalain). A subsequent impact survey conducted in Al Jabalain in 2015 and a surveillance survey in 2017 documented that the locality [17] had attained the World Health Organization’s (WHO) trachoma elimination threshold of TF less than 5% in children one to nine years and TT less than 0.2% in those 15 years and above [18]. Although all localities within White Nile state were considered non-endemic for trachoma as of 2015, given that many of the South Sudanese refugees were from endemic regions of South Sudan, the Federal Ministry of Health (FMOH) conducted baseline surveys in November 2017 in refugee camps located in Al Jabalain and Al Salam localities (Fig 1). The aim of these surveys was to estimate the prevalence of key trachoma indicators within South Sudanese refugee camps located in two Sudanese localities to determine whether SAFE strategy interventions were needed.

## Methods

### Ethics statement

Ethical clearance was received from the Sudan Federal Ministry of Health and the Emory University Internal Review Board (IRB #079–2006). Due to high illiteracy among the population, different languages of respondents and logistical constraints of written consent forms, IRB approval was obtained for verbal consent to be collected from all participants and recorded electronically. Verbal consent from a parent or guardian was required for those under 16 years of age. Participants were free to withdraw consent at any time without consequence.

### Setting

Most of the refugees in White Nile state are living in official refugee camps established by local authorities and international aid groups, while thousands of other displaced people live in informal settings [19]. Residents in the refugee camps are allowed to move outside the camps for work. Refugee camps located in Al Jabalain and Al Salam localities predominantly serve South Sudanese refugees and are managed by Sudan’s Commission of Refugees and White Nile State governmental departments, with support from non-governmental organizations. Official refugee camps within these two localities were surveyed to determine prevalence of trachoma. Informal camps and settlements were not included in the sampling frame as it would have been difficult to clearly separate from host community villages.

### Survey design

To estimate a TF prevalence of 3% in children aged one to nine years with a precision of 2%, given an assumed design effect of 3.0 and a 95% confidence level, a total sample size of 837 children was needed for each enumeration unit. Adjusting for an assumed non-response rate of 20% yields a target population size of 1,004 children per locality. Assuming 4.7 individuals per household and estimating that children aged one to nine years make up 35% of the population, a total of 611 households were targeted.

#### Al Salam refugee enumeration unit

**Fig 2:**
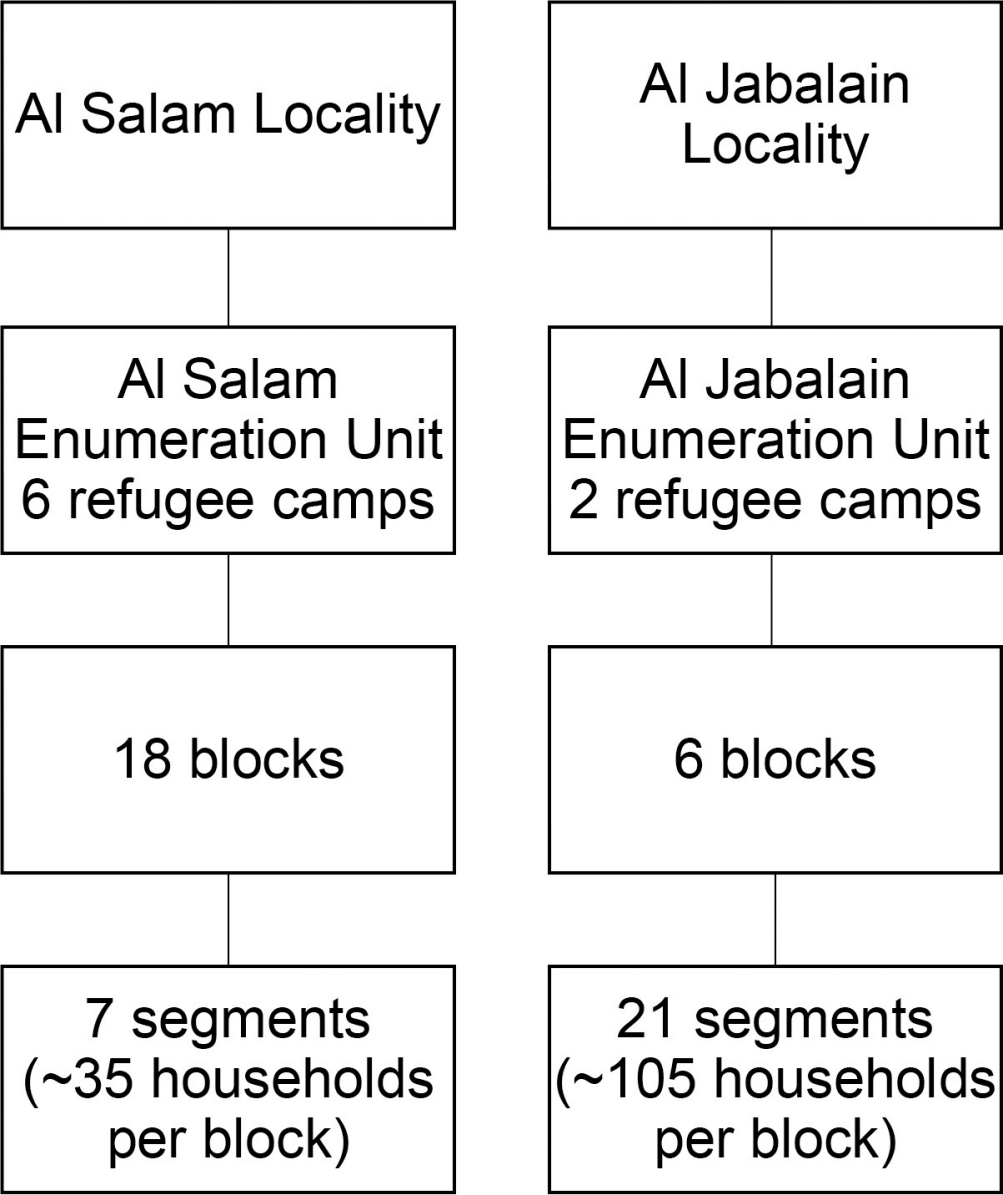
Sampling Frame, Al Salam Refugee and Al Jabalain Refugee enumeration units (EU).

All six official refugee camps within Al Salam locality (population = 125,083) were combined to form the sampling frame for that locality and will be referenced as Al Salam refugee enumeration unit (EU) (Fig 2). When refugee camps were established, camp administrators arranged households into “blocks” and numerated households with a serial number. This established structure of blocks was used as the cluster unit within the first stage of sampling. The list of blocks in each refugee camp and their estimated populations were obtained from the state level government. Given the unique nature of the refugee camp layout, we will continue to use the term “blocks” when referring to the clusters selected for analysis. All blocks across all refugee camps within Al Salam refugee EU were eligible for inclusion. To reach the required sample size, 18/47 (38%) blocks were selected randomly, with the number of blocks per camp selected according to the camp population size.

In the second stage, segmentation sampling was used to pick households. A household was defined as persons living together and eating from the same pot/sharing meals. A list of each household in selected blocks was created using camp records. Households were then grouped into segments of five households each and each segment was given a number. The number of each segment was written on a piece of paper and folded and placed in a bowl for random selection. For Al Salam refugee EU, seven segments (approximately 35 households) per block were randomly chosen by a local leader known by the community, and all households within these segments were interviewed and assessed.

#### Al Jabalain refugee enumeration unit

Because there were fewer camps in Al Jabalain locality, and each camp had fewer available blocks, the sampling plan was different compared to that of Al Salam. Both official refugee camps within Al Jabalain locality (population = 20,345) were combined to form the sampling frame and will be referenced as Al Jabalain refugee EU. To reach the required sample size, 6/12 (50%) blocks were selected randomly, with the number of blocks per camp selected according to the camp population size. In the second stage of sampling, households were segmented into five households each, and 21 segments (approximately 105 households) were randomly selected per block. All households within each segment were eligible for the survey.

### Training

Trachoma graders in Sudan are experienced Ophthalmic Medical Assistants. Prior to the surveys, graders participated in a three-day training on trachoma grading using the WHO simplified grading scheme [20]. This training consisted of classroom and field-based practice. All trainees were first required to pass a slide test of 50 standard trachoma images. Those that passed the slide test moved on to the field reliability exam. To pass the field reliability exam the trainees graded 50 eyes for trachoma signs at a field location. Trainees were then compared to the consensus grade of three expert graders including one designated master grader. Those trainees that achieved an 84% agreement or higher and a kappa of >0.7 when assessing the presence or absence of TF were allowed to move into the field for the survey.

All data recorders underwent a two-day training on how to use electronic tablets and survey software to collect data, conduct interviews, and properly select households and individuals to be interviewed following the standard protocol. All data recorders were required to pass an examination on their data collection skills to participate on the survey team.

### Data collection

Data collection occurred electronically on Samsung tablets using SWIFT Insights, a custom built application based on Open Data Kit [21]. After data collection finished, the survey data was transferred from the tablets’ micro SD cards to a computer via USB connection as deemed appropriate by Sudan’s national standards at the time. Teams were comprised of a trachoma grader, a data recorder, and a driver. One supervisor was responsible for two teams. The same five survey teams worked in each refugee EU. All residents of selected households were enumerated regardless of their presence and/or willingness to be examined. All present and consented children aged one to nine years were examined for TF, trachomatous inflammation-intense (TI), trachomatous scarring (TS) and TT, as defined by the WHO simplified grading scheme, using a 2.5X loupe and a flashlight [20]. All present and consented individuals ages 10 and older were examined for TT and corneal opacity (CO). Individuals determined to have TT had their eyelids flipped, if possible, and graded for signs of TS. A case of TT unknown to the health system was defined as an individual with at least one eye that has TT for which surgery has not been refused nor received. All children aged one to nine years were observed for an unclean face, defined as the presence of ocular or nasal discharge [22]. At the end of the day, survey teams made one follow-up attempt to return to homes to examine children aged one to nine years who were absent during the examination process. Households that were empty were not replaced by another household. Any child found to have TF or TI was provided with the antibiotic Zithromax in addition to the rest of the household. Those with TT were registered and counseled to have TT surgery during the next scheduled surgical campaign in their locality or camp.

### Household interview

Prior to the survey, the questionnaire was translated into Arabic and then back translated into English to ensure consistency of language. The structured interviews with adult household respondents were then conducted in Arabic at each selected household to assess demographic and household characteristics. In cases where the respondent did not speak or understand Arabic, a local translator from the camp was used. Adult females were given special preference for interviews as they are often the primary child caregivers and responsible for water collection and household chores. An improved water source was defined according to the WHO/UNICEF Joint Monitoring Programme for Water Supply and Sanitation and included protected dug well, protected spring, public tap, borehole, or piped water into dwelling [23]. Survey teams directly observed the presence of the latrines, and public and shared latrines were also considered household latrines. Questions also assessed face washing, time to fetch water (<30 minutes, 30–60 minutes, >60 minutes), level of adult education and household possessions, location of infant feces disposal, trachoma knowledge, access to electricity and mobile phones, and home state within South Sudan.

### Data analysis

Sampling weights were calculated as the inverse of the probability of selection at both stages of sampling. Variance estimates and confidence intervals were calculated using Taylor linearization through *svy* survey procedures in Stata 14.1 (STATA Corporation, College Station TX, USA). Maps were created using ArcGIS 10.6 (ESRI, Redlands CA, USA). All reported percentages with confidence intervals are weighted. The absence of males during the survey visit was perceived to be an issue because women carry an increased burden of TT compared to men [24]. Post-stratification weighting using five-year age-sex bands from the survey census population was used when estimating the prevalence of TT among those 15 years and older to account for the systematic absence among older males in this population.

## Results

From the 511 visited households in Al Salam refugee EU, 1,921 (81.6%) individuals of all ages were examined out of 2,355 individuals enumerated. Among children aged one to nine years, 916 (92.5%) individuals were examined out of 990 enumerated. Of the 397 households selected in Al Jabalain refugee EU, the response rate was 80.3% (1,430 individuals examined /1,781 enumerated) among all ages and 89.8% among children aged one to nine years (685 examined / 763 enumerated). In both refugee EU surveys, females were more likely than males to be examined for trachoma. Children aged one to nine years made up nearly 48% of the examined population (Table 1).

**Table 1:** Demographics of examined participants, White Nile State, Sudan, 2017.

Characteristic*	Al Salam N(%)	Al Jabalain N(%)
Sex		
Male	640 (33.4%)	613 (43.1%)
Female	1,276 (66.6%)	810 (56.9%)
Age group, years		
1–9	916 (47.7%)	685 (47.9%)
10–19	468 (24.4%)	321 (22.5%)
20–29	192 (10.0%)	143 (10.0%)
30–39	134 (7.0%)	106 (7.4%)
40–49	91 (4.7%)	66 (4.6%)
50–59	24 (1.3%)	24 (1.7%)
60–69	36 (1.9%)	26 (1.8%)
70+	51 (2.7%)	43 (3.0%)

* Where data was missing, missing data for each variable for combined evaluation units were as follows: sex (n = 13); age (n = 7).

In regards to water, sanitation and hygiene indicators, nearly all households had access to an improved water source and could collect water within 30 minutes or less roundtrip (Table 2). The great majority of adult household respondents reported that the children had their face washed at least once a day. Upon direct observation at the time of the survey, facial cleanliness among children aged one to nine years was nearly 73% in both camps. The observed prevalence of a latrine was considerably higher in Al Salam refugee EU, 99.7% (95%CI: 97.3–100.0), compared to Al Jabalain refugee EU, 70.9% (95%CI: 51.4–84.9). Perhaps because of this difference, household respondents reported that they disposed of their children’s excretion in a latrine more often in Al Salam than in Al Jabalain. Although household electricity was scarce or nonexistent in the camps, a majority of households in both camps reported having a mobile phone. Trachoma knowledge was low in both camps, but among those who knew of trachoma, facial and environmental cleanliness were the most commonly reported ways to prevent trachoma (Table 3). A very small percentage of household respondents reported knowing that surgery can protect someone from trachoma.

**Table 2:** Individual and household characteristics in two refugee evaluation units, White Nile State, Sudan, 2017.

Characteristics*	Al Salam % (95%CI)	Al Jabalain % (95%CI)
**Individual**		
Children ages 1–9 years with clean face (observed)	72.7 (65.6–78.9)	72.8 (65.2–79.3)
**Household**		
Caregivers washing children’s faces		
Never	0.0 (0.0–0.0)	0.3 (0.0–2.9)
Every few days	2.6 (1.3–5.4)	2.0 (0.6–6.4)
Once a day	21.1 (14.1–30.2)	20.3 (16.1–25.3)
Twice a day	47.5 (39.7–55.4)	52.4 (44.9–59.7)
More than twice a day	28.9 (20.4–39.1)	25.1 (19.8–31.1)
Presence of latrine (observed)	99.7 (97.3–100.0)	70.9 (51.4–84.9)
Disposal of children’s excretion		
Bury it in place	16.3 (10.4–24.6)	12.0 (8.6–16.6)
Collect it and dispose in latrine	42.7 (28.9–57.7)	18.6 (9.8–32.4)
Leave it as it is	5.7 (3.7–8.8)	2.2 (1.1–4.5)
Collect and throw in an open area	35.3 (25.3–46.8)	67.2 (58.8–74.6)
Improved primary source of water	99.2 (97.5–99.7)	95.0 (90.5–97.4)
Time to collect water		
<30 minutes	97.0 (94.1–98.5)	96.9 (94.7–98.2)
30–60 minutes	3.0 (1.5–5.9)	2.9 (1.6–5.0)
>60 minutes	0.0 (0.0–0.0)	0.2 (0.0–0.8)
Any adult education	81.2 (75.6–85.8)	66.9 (62.6–70.9)
Household possessions		
Functioning household electricity	0.1 (0.0–1.2)	0.0 (0.0–0.0)
Radio ownership	14.7 (9.7–21.7)	46.0 (37.2–55.1)
Mobile phone ownership	95.8 (92.6–97.7)	95.4 (92.3–97.3)

*Where data was missing, missing data for each variable for combined evaluation units were as follows: caregivers washing children’s faces (n = 11); presence of latrine (n = 18); improved primary source of water (n = 4); time to collect water (n = 6); any adult education (n = 9); household possessions (n = 248).

**Table 3:** Trachoma knowledge and awareness, White Nile State, Sudan, 2017.

Characteristics*	Al Salam % (95%CI)	Al Jabalain % (95%CI)
Has heard of trachoma	22.1 (15.6–30.5)	17.6 (14.0–21.9)
How can someone be protected from trachoma**		
I do not know	42.7 (30.3–56.1)	46.1 (29.9–63.2)
Surgery	0.6 (0.0–4.7)	0.0 (0.0–0.0)
Antibiotics	2.3 (0.4–13.8)	6.1 (2.1–16.4)
Face washing	33.6 (24.7–43.8)	23.8 (11.9–41.9)
Keeping environment clean	14.0 (7.6–24.2)	21.0 (11.8–34.6)
Other	6.9 (23.4–18.5)	3.0 (0.5–15.6)

*Where data was missing, missing data for each variable for combined evaluation units were as follows: heard of trachoma (n = 1); How can someone be protected from trachoma (n = 47).

**Prevalence among those who reported having heard of trachoma

**Fig 3:**
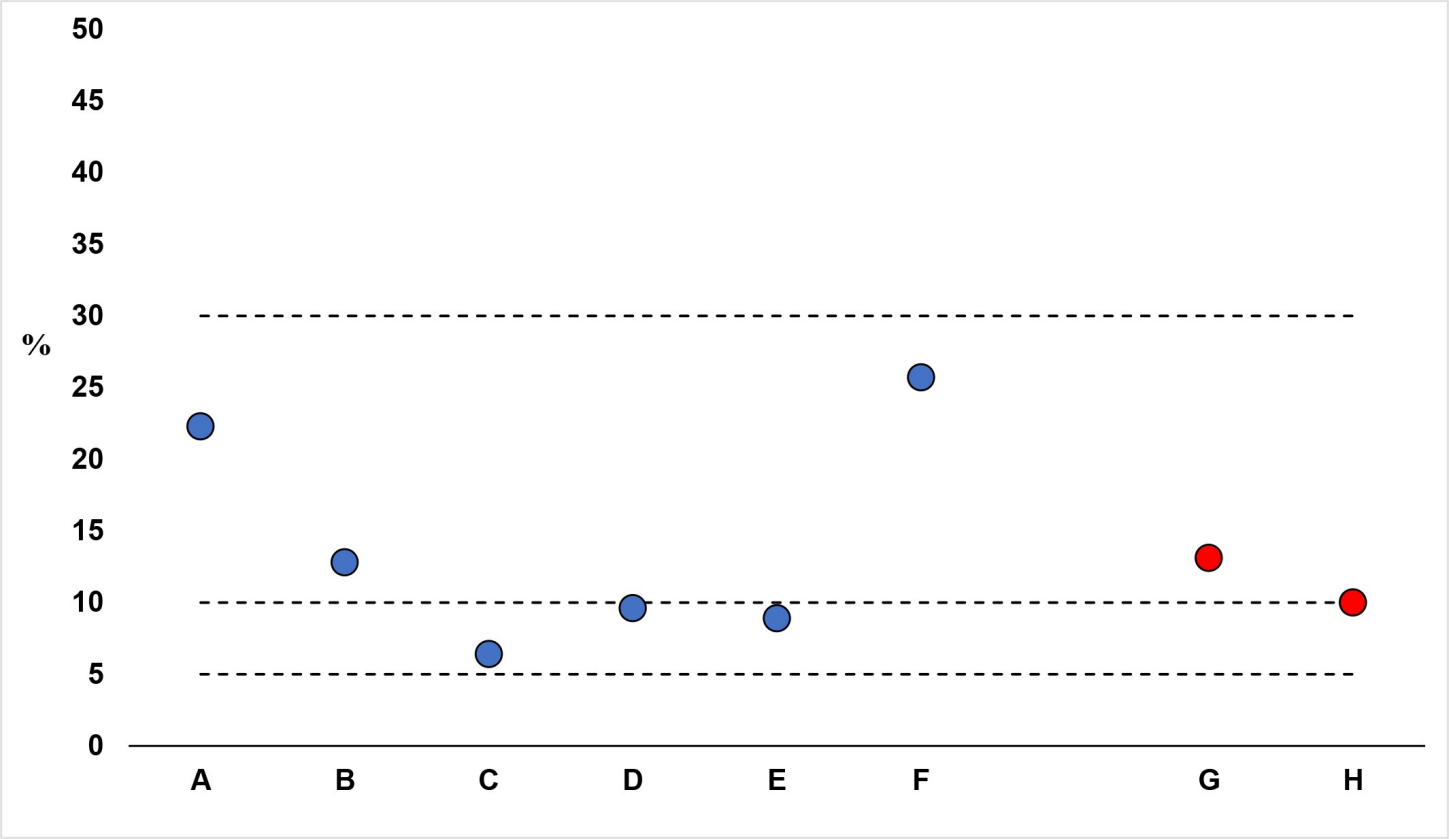
Prevalence of TF among children ages one to nine years by camp (weighted), in Al Salam (Camps A-F) and Al Jabalain (Camps G, H) refugee camps. * Dashed lines represent WHO guidelines for the SAFE strategy 5–9.9%, 1 year MDA, 10–29.9% 3 years MDA, ≥30% 5 years MDA.

The prevalence of TF in children aged one to nine years was 15.7% (95%CI: 12.1–20.2) in Al Salam refugee EU and 10.6% (95%CI: 7.9–14.0) in Al Jabalain refugee EU (Table 4). Within each individual camp, the prevalence of TF was above the 5% elimination threshold, with most having a prevalence of 10% or greater (Fig 3). In Al Salam refugee EU and Al Jabalain refugee EU, the prevalence of TI among children aged one to nine years was very low, and no TS was observed in this age group. The prevalence of TT in those aged 15 years and above exceeded the elimination threshold of 0.2% in both EUs (5.0% (95%CI: 3.8–6.6) in Al Jabalain and 2.9% (95%CI: 1.8–4.8) in Al Salam). The prevalence of CO was rare in both refugee EUs.

**Table 4:** Prevalence of clinical signs of trachoma in two refugee evaluation units, White Nile State, Sudan, 2017.

Clinical Sign	Al Salam % (95%CI)	Al Jabalain % (95%CI)
TF, ages 1-9y	15.7 (12.1–20.2)	10.6 (7.9–14.0)
TI, ages 1-9y	0.2 (0.0–0.8)	0.0 (0.0–0.0)
TF and/or TI, ages 1-9y	15.7 (12.1–20.2)	10.6 (7.9–14.0)
TT, ages ≥15y†	2.9 (1.8–4.8)	5.0 (3.8–6.6)
TT, ages ≥15y*†	1.8 (1.0–3.2)	4.8 (3.6–6.4)
TT, all ages*	0.8 (0.5–1.4)	2.0 (1.5–2.6)
CO, ages ≥ 15y	0.2 (0.0–0.9)	0.0 (0.0–0.0)

* TT unknown to health system

†Post-stratified based on 5-year age-sex bands. TF = trachomatous inflammation- follicular; TI = trachomatous inflammation-intense; TT = trachomatous trichiciasis; CO = corneal opacity

**Fig 4:**
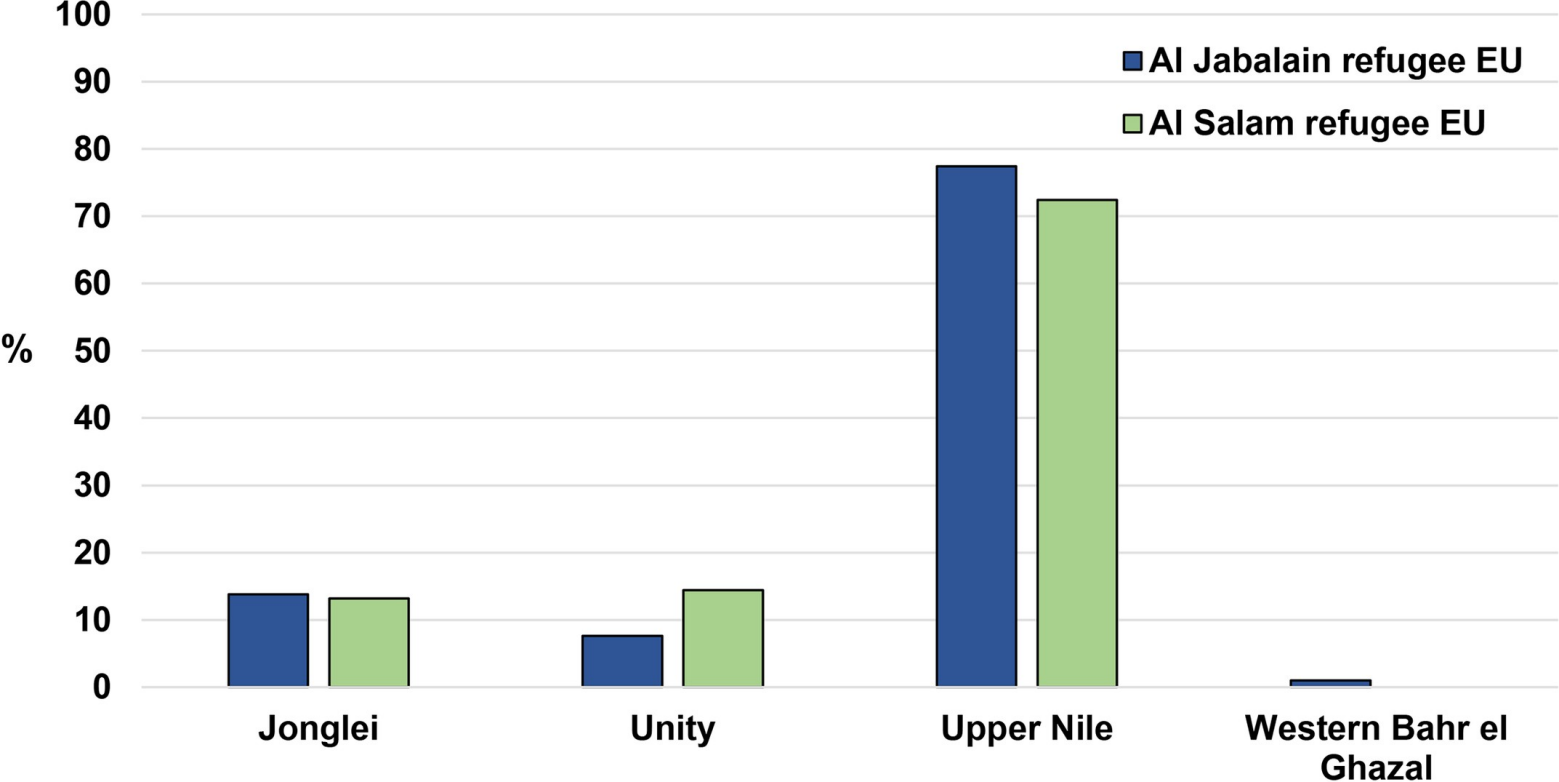
Self-reported South Sudan home state of refugees, White Nile State, Sudan, 2017.

Participants in these two refugee EUs reported coming from four states in South Sudan. The most commonly reported state was Upper Nile state for both Al Salam (72.4%) and Al Jabalain (77.4%) refugee EUs. A considerable proportion of individuals reported coming from Unity and Jonglei states, and just a few in Al Jabalain refugee EU reported Western Bahr el Ghazal as their home state (Fig 4).

## Discussion

The results of these population-based surveys demonstrated endemic levels of trachoma in refugee camps located within host localities considered to have eliminated trachoma as a public health problem. Within both refugee EUs, the prevalence of TF among children aged one to nine years was greater than 10% and the prevalence of TT in those 15 years and older was considerably greater than 0.2%. The FMOH of Sudan should conduct SAFE interventions in all surveyed camps that include three rounds of MDA and the provision of surgical services to avoid TT-related blindness. The current global trachoma elimination effort coincides with a period of record level human displacement. Trachoma control programs serving areas which host displaced populations should consider the possibility that timely elimination of trachoma as a public health problem may be threatened if these populations do not receive quality SAFE interventions.

The South Sudanese populations surveyed in these refugee camps came predominately from Upper Nile, Unity, and Jonglei states of South Sudan. Though baseline mapping in South Sudan is incomplete, what has been surveyed showed that Upper Nile, Unity, and Jonglei states were hyper-endemic for trachoma [9,10,13] with one district having a prevalence as high as 80.1% TF in children and 14.6% TT in adults 15 years and above [10]. Many of the districts surveyed had not received SAFE interventions since baseline surveys were conducted. Due to insecurity, mass displacement of populations and a lack of funding, all other districts that were implementing the SAFE program had to suspend activities [25]. SAFE activities have yet to resume in the three states as of December 2018. Not only have activities been suspended in areas that are known to be endemic, but it has been difficult to finish baseline surveys in other suspected endemic districts in the region. Given the suspended program in the northern region of South Sudan, the known level of trachoma endemicity there, and that refugee camp residents originated from these three states, it is unsurprising that the documented trachoma prevalence levels were greater than 10% TF in children aged one to nine years and TT levels greater than 2.9% in adults 15 years and above within the camp surveys.

Changing population dynamics associated with displaced populations, whether IDPs or refugees, is an important issue for countries working to document their success in achieving WHO trachoma elimination targets. In these situations, country programs must determine how to address both the displaced populations and the hosting communities. Although there is sparse research on the effect of human displacement on trachoma control, it is clear that civil conflict and poverty can lead to increased transmission of infectious diseases including NTDs [26]. Furthermore, it has been demonstrated that forced or voluntary migration can also lead to the reintroduction of NTDs, including the reintroduction of Chlamydial infection, into communities where the prevalence was previously low [27,28]. It is still to be determined, however, if reintroduction of trachoma infection locally can result in a non-endemic district developing sustained trachoma levels above the elimination threshold [29]. In White Nile State, all localities were either non-endemic for trachoma or had reached the elimination target following sustained SAFE interventions. Refugee camps, however, were not included in the sampling frame of those earlier surveys in Al Jabalain or Al Salam localities. In the case of Al Salam locality, refugee camps house over 125,000 residents, a population figure that is greater than some locality population sizes in Sudan. This refugee population now has a demonstrated prevalence of 15.7% TF. Depending on the degree of mixing with the population outside the camp, there is a concern of reemerging Chlamydial infection in Al Salam locality more broadly. Comprehensive SAFE interventions, as well as the documented WASH indicators in the camps themselves may help contain and eliminate trachoma from these camps before it becomes a larger problem.

Displaced populations, like permanent residents living in NTD endemic locations, often have multiple health challenges that need to be addressed. The objectives of humanitarian action include saving lives, alleviating suffering and maintaining human dignity [30]. Populations living in trachoma endemic refugee and IDP camps are at risk of continued infection with trachoma which could ultimately lead to blindness. Those who have trachoma have the right to be provided with treatment (whether through MDA or surgery) and education on how to prevent the disease. Providing SAFE services to refugees and IDPs while they are living in the camps not only reduces their risk of going blind but can provide other positive health outcomes. Azithromycin has been shown to decrease mortality among children under five years of age, reduce diarrhea and is the recommended line of therapy for pediatric patients with cholera [31–33]. Additionally, an increased focus on WASH related behaviors such as latrine use and personal hygiene can contribute to reduction in other non-trachoma diseases [34].

Implementing NTD programs within refugee and IDP settings is not without challenges. Often barriers to providing NTD services to refugees and IDPs exist: national NTD programs have limited resources for the host population, methods for obtaining MDA drugs are unclear, refugee care may fall under a separate ministry or legal institutions, and NTD supporting organizations may have difficulty gaining access to refugees because they are outside of their normal village-based programming [26]. Additionally, population dynamics within the camps vary. Displaced populations are increasingly composed of more children, elderly and people with disabilities [35]. A report from UNHCR reported that the majority (82%) of South Sudanese refugees in Sudan are women and children, a finding similar to that reported in our surveys [7]. Furthermore, these surveys showed that there were more children per household (48%) than typically found in non-refugee settings within Sudan (35%). This may be due to parents sending their children out of harm’s way or orphans staying with relatives residing in camps. These child-related dynamics must be taken into consideration by drug donation programs when determining the percentage of drug needed by children. These challenges highlight the need for governments and supporting organizations to document and share lessons learned. Preferred practices should also be developed in consultation with governments, UN agencies and implementing organizations, both from the NTD and humanitarian response sectors.

Recognizing the challenges of providing NTD programs to refugees and IDPs, it is therefore important to more closely align public health activities, such as trachoma elimination, with the humanitarian agenda aimed at these populations. In the case of South Sudanese refugees in Sudan this need for alignment is especially important as the states where the refugees originate are still largely insecure and inaccessible to the Republic of South Sudan NTD program. WHO’s Expanded Special Project for Elimination of NTDs (ESPEN) considers South Sudan one of the five priority countries in the African region with the highest burden of NTDs [36]. Taking advantage of opportunities to provide NTD related services to South Sudanese known to be affected with NTDs, regardless of their physical location, helps the global program achieve its targets. By providing services to refugees where they are, while waiting to gain access to where they came from, ensures affected populations are not neglected while they wait to return home.

As demonstrated by an earlier trachoma survey conducted in Ethiopia, the ‘block’ nature of the camps allowed for easy selection and compact household locations made logistics easier and took less time to survey [37]. Applying these methods, we have shown that it is possible to conduct trachoma prevalence surveys within refugee camp settings in Sudan and that the information can be used to plan for SAFE strategy interventions. Though planning and implementing a survey within camps required different levels of engagement with the locality, state, and administrative units that govern the camps, once access was granted, the structured nature of the camps made it easier than a typical district survey. Safety was not a concern. Though the structure of the camps made some aspects of surveys easier, there were also challenges and limitations. Camp-specific daily and monthly travel patterns should be considered when planning surveys as this can impact the number of people home at the time of the survey. Al Jabalain refugee EU survey team did not reach its targeted number of children, possibly due to timing of the survey where households were empty as residents were attending food distributions or assisting in food cultivation in farms. This may have reduced the precision of the TF estimate in that EU. Not all camp residents spoke Arabic; therefore, in some cases, a local guide would have to translate the questions and answers into the respondent’s native language. Data on how long camp residents had been in the camp prior to the survey was not collected. This information could have been useful in better gauging whether clinical signs of trachoma was due to recent exposure in South Sudan or newly acquired from Sudan. It is therefore suggested this information is collected in future surveys if possible.

As Sudan continues to make progress in reducing trachoma in formerly endemic localities such as Al Jabalain, the FMOH has shown forethought in anticipating the possible impact of hosting large communities of refugees originating from trachoma endemic countries. By beginning the process of conducting baseline surveys in the camps now, before the country begins working on the WHO dossier to validate trachoma elimination as a public health problem, the trachoma control program has been able to better understand prevalence dynamics and plan program interventions. Following these 2017 refugee baseline surveys, in 2018 the FMOH conducted TT surgical outreaches within the camps. During the outreach, health education about how to prevent and treat trachoma was provided. Moving forward, the Sudan FMOH should conduct three rounds of MDA in addition to surgical campaigns and health education within the surveyed camps. Following three years of intervention, an impact survey should be conducted to monitor progress in reducing TF and TT within the population. Additionally, follow-up surveys should be considered for Al Salam and Al Jabalain at the locality level to confirm that the presence of the refugee population has not impacted the prevalence of trachoma within the locality. Given the findings of these surveys in White Nile state, the FMOH should also consider conducting baseline mapping in other South Sudanese refugee camps [8] where residents are suspected to have originated from trachoma endemic regions of South Sudan.

A recently published systematic review of visual health in refugees concluded that there were no studies assessing the state of eye health in refugee groups from recent or current conflicts [38]. Governments and supporting organizations are beginning to fill this knowledge gap in countries such as Ethiopia, Uganda and Sudan, all countries hosting South Sudanese refugees [37,39–41]. The global community cannot expect to achieve the goals set out in the 2012 London Declaration on NTDs if it does not take into consideration the impact of conflict on programs and the increasing displacement of populations from endemic regions into endemic and non-endemic regions [42,43]. We found that there were refugee EUs with trachoma that require SAFE interventions in two non-endemic Sudanese localities. These groups must be accounted for during routine planning, implementation and surveillance activities if countries aim to be validated as eliminating trachoma as a public health problem within their borders.

## Supporting information

S1 Checklist STROBE checklist. (DOC)
