# Supplementary material for: Prevalence of trachoma within refugee camps serving South Sudanese refugees in White Nile State, Sudan: Results from population-based surveys
Source: PLoS Negl Trop Dis. 2019 Jun 13;13(6):e0007491. doi: 10.1371/journal.pntd.0007491 (PMC6592575; doi:10.1371/journal.pntd.0007491)
Supplement: S1 Checklist — (DOC) [file pntd.0007491.s001.doc]

STROBE Statement—Checklist of items that should be included in reports of ***cross-sectional studies***

|  | Completed  Yes/ No | Item No | Recommendation |
| --- | --- | --- | --- |
| **Title and abstract** | Yes | 1 | (*a*) Indicate the study’s design with a commonly used term in the title or the abstract  *The title describes the study design as “population based surveys”* |
|  | Yes |  | (*b*) Provide in the abstract an informative and balanced summary of what was done and what was found  *The abstract describes the methods and findings.* |
|  | Introduction | | |
| Background/rationale | Yes | 2 | Explain the scientific background and rationale for the investigation being reported  *The background and rational are described in the Introduction, paragraphs 1, 2, &4* |
| Objectives | Yes | 3 | State specific objectives, including any prespecified hypotheses  *The study design is discussed in paragraph 1 of the Introduction and in the ‘survey design’ sub-section of the methods section* |
|  | Methods | | |
| Study design | Yes | 4 | Present key elements of study design early in the paper  *The study design is discussed in the ‘survey design’ sub-section of the methods section* |
| Setting | Yes | 5 | Describe the setting, locations, and relevant dates, including periods of recruitment, exposure, follow-up, and data collection  *The programmatic setting, location and dates of survey are described in the ‘setting’ sub-section of the Methods section.*  *The training and data collection are described in the ‘training’ and ‘data collection’ sub-sections of the Methods section.* |
| Participants | Yes | 6 | (*a*) Give the eligibility criteria, and the sources and methods of selection of participants  *Selection of sample and eligibility criteria are discussed in the ‘data collection’ sub-section of Methods section* |
| Variables | Yes | 7 | Clearly define all outcomes, exposures, predictors, potential confounders, and effect modifiers. Give diagnostic criteria, if applicable  *Outcomes for clinical signs of trachoma are defined in the data collection paragraph of the method section.* |
| Data sources/ measurement | Yes | 8* | For each variable of interest, give sources of data and details of methods of assessment (measurement). Describe comparability of assessment methods if there is more than one group  *Measurements of outcomes (clinical signs of trachoma) are discussed in the ‘data collection’ sub-section of the Methods section; Demographic and household characteristics are described in ‘household interview’ sub-section of Methods section.* |
| Bias | Yes | 9 | Describe any efforts to address potential sources of bias  *Addressed through training conducted as discussed in paragrapsh 6 and 7 in Methods section.* |
| Study size | Yes | 10 | Explain how the study size was arrived at  *Sample determination is discussed in ‘survey design’ sub-section in Methods section* |
| Quantitative variables | Yes | 11 | Explain how quantitative variables were handled in the analyses. If applicable, describe which groupings were chosen and why  *Use of variables is dicussed in ‘data analysis’ sub-section of Methods section.* |
| Statistical methods | Yes | 12 | (*a*) Describe all statistical methods, including those used to control for confounding  *Statistical methods discussed in ‘data analysis’ sub-section of Methods section* |
|  | Yes |  | (*b*) Describe any methods used to examine subgroups and interactions  *Statistical methods discussed in ‘data analysis’ sub-section of Methods section* |
|  | Yes |  | (*c*) Explain how missing data were addressed  *Statistical methods discussed in ‘data analysis’ sub-section of Methods section* |
|  | Yes |  | (*d*) If applicable, describe analytical methods taking account of sampling strategy  *Statistical methods discussed in ‘data analysis’ sub-section of Methods section* |
|  | Not applicable |  | (*e*) Describe any sensitivity analyses |
|  | Results | | |
| Participants | Yes | 13* | (a) Report numbers of individuals at each stage of study—eg numbers potentially eligible, examined for eligibility, confirmed eligible, included in the study, completing follow-up, and analysed  *Individuals at each stage of study are described in first paragraph in Results section.* |
|  | Yes |  | (b) Give reasons for non-participation at each stage  *Addressed in paragraph 7 of Discussion section* |
|  | Yes |  | (c) Consider use of a flow diagram  *Figure 2. Sampling Frame, Al Salam Refugee and Al Jabalain Refugee enumeration units (EU)* |
| Descriptive data | Yes | 14* | (a) Give characteristics of study participants (eg demographic, clinical, social) and information on exposures and potential confounders  *Table 1: Demographics of examined participants, White Nile State, Sudan, 2017.*  *Table 2: Individual and household charactersitics in two refugee evaluation units, White Nile State, Sudan 2017* |
|  |  |  | (b) Indicate number of participants with missing data for each variable of interest  *Paragraph 1 in Results section reports response rate and Tables 1 and 2: indicate missing values for variables* |
| Outcome data | Yes | 15* | Report numbers of outcome events or summary measures  *Numbers and precentages are reported througouth the Results Section* |
| Main results | Yes | 16 | (*a*) Give unadjusted estimates and, if applicable, confounder-adjusted estimates and their precision (eg, 95% confidence interval). Make clear which confounders were adjusted for and why they were included  *Table 2: Individual and household charactersitics in two refugee evaluation units, White Nile State, Sudan 2017*  *Table 3: Trachoma knowledge and awareness, White Nile State, Sudan 2017*  *Table 4: Prevalence of clinical signs of trachoma in two refugee evaluation units, White Nile State, Sudan 2017*  *All results weighted for survey design, as noted in Data analysis paragraph, further adjustments noted in table footnotes if applicable.* |
|  | Not applicable |  | (*b*) Report category boundaries when continuous variables were categorized |
|  | Not applicable |  | (*c*) If relevant, consider translating estimates of relative risk into absolute risk for a meaningful time period |
| Other analyses | Not applicable | 17 | Report other analyses done—eg analyses of subgroups and interactions, and sensitivity analyses |
|  | Discussion | | |
| Key results | Yes | 18 | Summarise key results with reference to study objectives  *Results are summarized in first parapgraph in Discussion section* |
| Limitations | Yes | 19 | Discuss limitations of the study, taking into account sources of potential bias or imprecision. Discuss both direction and magnitude of any potential bias  *Discussed in pargraph 7 of Discussion* |
| Interpretation | Yes | 20 | Give a cautious overall interpretation of results considering objectives, limitations, multiplicity of analyses, results from similar studies, and other relevant evidence  *Discussed in paragpraphs 1 through 3 in Discussion section* |
| Generalisability | Yes | 21 | Discuss the generalisability (external validity) of the study results  *Discused in paragraphs 3, 4, 5, 6, and 9 in Discussion section* |
|  | Other information | | |
| Funding | Yes | 22 | Give the source of funding and the role of the funders for the present study and, if applicable, for the original study on which the present article is based  *Discussed in Funding section* |

*Give information separately for exposed and unexposed groups.

**Note:** An Explanation and Elaboration article discusses each checklist item and gives methodological background and published examples of transparent reporting. The STROBE checklist is best used in conjunction with this article (freely available on the Web sites of PLoS Medicine at http://www.plosmedicine.org/, Annals of Internal Medicine at http://www.annals.org/, and Epidemiology at http://www.epidem.com/). Information on the STROBE Initiative is available at www.strobe-statement.org.
